# *Aedes aegypti* Mosquitoes from Central Vietnam Feature Specific Viromic Profiles Linked to Dengue Virus Coinfection

**DOI:** 10.3390/v18040422

**Published:** 2026-03-31

**Authors:** Margarita Popova, Alena Khalilova, Anna Gladkikh, Ekaterina Klyuchnikova, Tatiana Arbuzova, Edward Ramsay, Nguyen T. Dong, Bui T. Phu, Hung Thai Do, Vladimir Dedkov

**Affiliations:** 1 Saint Petersburg Pasteur Institute, Saint Petersburg 197101, Russia; 2 Pasteur Institute in Nha Trang, Nha Trang 650000, Vietnam; 3 Martsinovsky Institute of Medical Parasitology, Tropical and Vector Borne Diseases, Sechenov First Moscow State Medical University, Moscow 119048, Russia

**Keywords:** *Aedes aegypti*, dengue virus, insect-specific viruses (ISVs), metavirome, Vietnam

## Abstract

*Aedes aegypti* is a primary vector for globally significant arboviruses such as dengue virus (DENV). The mosquito’s metavirome, particularly its insect-specific virus (ISV) component, is recognized as a key modulator of arboviral transmission. However, the natural ecology of these interactions in populations remains poorly understood. This study presents the first comparative analysis of the metavirome in wild-caught *A. aegypti* from Vietnam based on natural DENV infection status. Metaviromic analysis was performed on 69 DENV-positive pools from six central provinces. The results obtained were compared with previously obtained metaviromic data from 7 DENV-negative pools (from the same region). Analysis suggests the presence of a stable ‘core metavirome’ of 11 ISVs present in both groups. Interestingly, six ISVs were detected only in DENV-negative mosquitoes, which may suggest potential antagonistic interactions requiring further investigation. Conversely, five ISVs were found only in DENV-positive pools, including *Aedes partiti-like virus 1* and *Aedes anphevirus*. The latter may suggest possible synergistic relationships that facilitate arboviral replication. Phylogenetic analysis of prevalent ISVs, such as *Phasi Charoen-like phasivirus* (PCLV) and *Chaq-like virus*, revealed patterns of both local circulation and genetic diversity. The findings describe distinct ISV profiles associated with DENV infection in a natural setting, providing a data-driven foundation for hypothesizing specific virus–virus interactions. The data underscores the complexity of the mosquito metavirome. Here, we identified several candidate ISVs for future experimental studies aimed at understanding potential functional impact on arboviral vector competence.

## 1. Introduction

*Aedes* mosquitoes, particularly the ubiquitous urban adapter *Aedes aegypti* (*A. aegypti*), function as primary vectors for a group of medically significant arboviruses. This group includes dengue (DENV), Zika (ZIKV), chikungunya (CHIKV), and yellow fever (YFV) viruses, whose global spread poses a persistent public health challenge [1]. The burden of these pathogens is starkly illustrated by dengue. The World Health Organization estimates 100–400 million dengue infections annually, solidifying its status as the most prevalent mosquito-borne viral disease globally [2].

In recent decades, research focus has shifted from exclusively pathogenic arboviruses to the complex and diverse mosquito metavirome, a key component of which is insect-specific viruses (ISVs). These viruses, whose replication is restricted to insect cells, are non-infectious to vertebrate hosts [3]. Most importantly, accumulated data strongly indicate that ISVs can modulate the replication of pathogenic arboviruses during coinfection. This modulation can profoundly impact vector competence. Some ISVs act as transmission suppressors, while others may paradoxically enhance it [4,5]. Thus, the mosquito metavirome represents not merely a passenger community, but a critical factor determining the dynamics of arboviral transmission.

Despite progress achieved in laboratory settings, the ecology and functional roles of ISVs in natural mosquito populations remain insufficiently studied. A key unresolved challenge is understanding the bidirectional nature of the interaction between the resident ISV metavirome and an invasive arbovirus like DENV in field conditions. Our previous research established a crucial baseline by characterizing the ISV diversity in DENV-uninfected *A. aegypti* mosquitoes from Binh Thuan province, Vietnam, providing the first snapshot of viral diversity in this endemic region [6].

Building directly on these data, the present study aims not only to investigate the mosquito metavirome, but also to conduct a comparative metaviromic analysis of wild-caught *A. aegypti* from the same region to identify explore potential differences in insect-specific virus (ISV) composition between individuals naturally infected with DENV (DENV-positive) and their DENV-negative counterparts. The findings help describe potential associations between specific ISVs and DENV infection under natural conditions. Such analysis is an important step in understanding the ecology of viral co-circulation in vector populations. Prospectively, the identified patterns may serve as a basis for generating hypotheses about functional virus–virus interactions within the mosquito. Such patterns may also help identify potential ISV candidates for further investigation regarding their impact on vector competence.

## 2. Materials and Methods

### 2.1. Study Design and Sample Characterization

**Figure 1:**
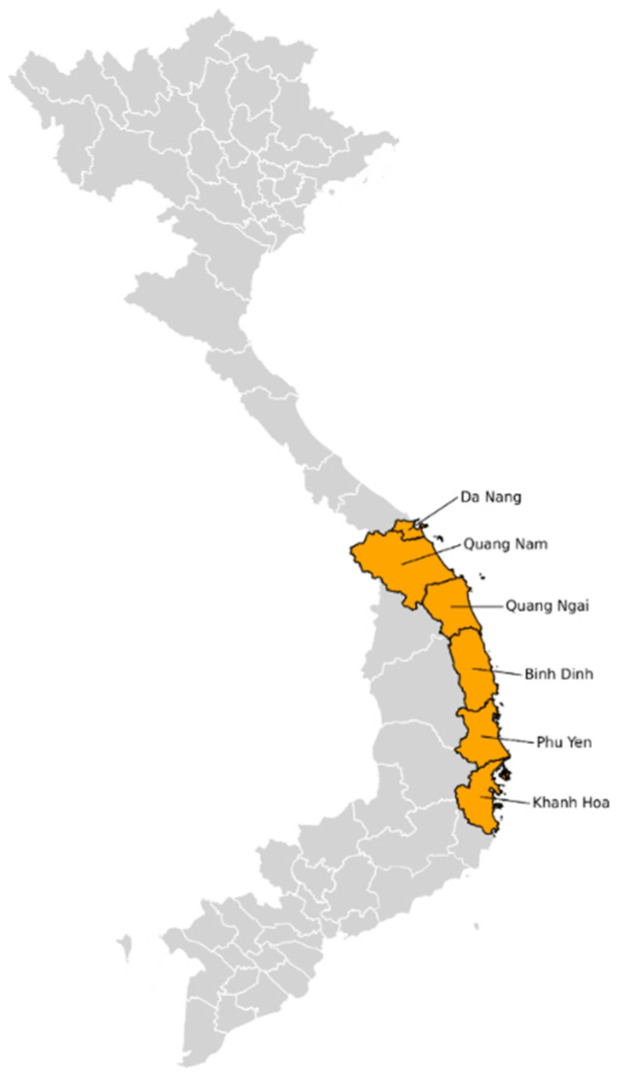
Map of sampling locations. Vietnamese provinces where samples collected. The GADM v4.0, GeoPandas v1.0.1, and Matplotlib v3.7.0 [8] packages were used.

The study was conducted using mosquito samples obtained within the framework of the dengue virus (DENV) epidemiological surveillance program in Vietnam. This monitoring effort assembled an extensive collection of *A. aegypti* mosquito pools collected throughout 2023, a subset of which tested positive for DENV as previously described [7]. The sample set for the present investigation was purposefully selected from this collection. All 69 mosquito pools (representing 308 mosquitoes) that had tested positive for DENV via real-time RT-PCR were chosen for in-depth metaviromic analysis. These pools were collected across six provinces in Central Vietnam: Da Nang, Quang Nam, Quang Ngai, Binh Dinh, Phu Yen, and Khanh Hoa (Figure 1). Each pool contained from 2 to 10 individuals, grouped by metadata (species, location, collection date). The detailed distribution of samples across provinces is provided in the Supplementary Materials (Table S1).

To assess potential differences in metaviromic profile associated with DENV infection, data from the present study were compared with metaviromic data from uninfected *A. aegypti* mosquitoes obtained in our previous study conducted in the same general region (based on the analysis of 7 mosquito pools from Binh Thuan province). The similarity in methodologies at specific stages (processing, sequencing, bioinformatics) enables such a comparison and permits interpretation of the identified differences in the context of the impact of DENV infection [6].

### 2.2. Preparation of Mosquito Samples, RNA Extraction, PCR

Each selected mosquito pool was homogenized for subsequent RNA extraction and viral analysis. Collected samples were placed in 70% ethanol to remove surface contaminants and rinsed twice in a sterile phosphate-buffered saline solution (PBS, Sigma-Aldrich, St. Louis, MO, USA). Each mosquito pool was homogenized in 2 mL microcentrifuge tubes (Eppendorf, Hamburg, Germany) with stainless steel beads (QIAGEN, Hilden, Germany) and 500 microliters (μL) of 1× PBS (Sigma-Aldrich, St. Louis, MO, USA). Homogenization was performed using a Tissue Lyser LT homogenizer (QIAGEN, Hilden, Germany). Supernatant (450 μL) was transferred into 2 mL microcentrifuge tubes for further RNA isolation and storage.

Nucleic acids from mosquito samples were analyzed for the presence of DENV using the Dengue virus-FL RT-PCR kit (AmpliSens, Moscow, Russia). RT-PCR was performed using a CFX96 C1000 Touch TM instrument (Bio-Rad, Hercules, CA, USA). These samples were then used for metaviromic sequencing.

### 2.3. Library Preparation

Libraries for metaviromic sequencing were prepared from the extracted RNA using the KAPA RNA HyperPrep Kit (Roche, Mannheim, Germany) according to the manufacturer’s protocol. The procedure included RNA fragmentation, cDNA synthesis, A-tailing, and adapter ligation (KAPA Universal Adapter, Roche, Mannheim, Germany). Post-ligation clean up and post-amplification clean up were performed using KAPA Pure Beads (Roche, Mannheim, Germany).

Library quality was assessed using the QIAxcel Advanced System (QIAGEN, Germany), confirming a fragment size distribution of 190–450 bp with a median of 220–270 bp. Quantification was performed using a Qubit 4.0 Fluorometer and the Qubit dsDNA HS Assay Kit (Thermo Fisher Scientific, Waltham, MA, USA). The pooled libraries were sequenced on the Illumina MiSeq platform (Illumina, San Diego, CA, USA) using a MiSeq Reagent Kit v3 for 2 × 300 bp paired-end reads. A negative control (no-template control) was included during library preparation and sequenced alongside the samples to assess potential cross-contamination.

### 2.4. Contig Assembly and BLAST Analysis

Raw sequencing reads were subjected to quality control using FastQC (v0.12.1) [9]. Adapters and low-quality sequences were trimmed using Trimmomatic (v0.39) with a sliding window of 4 bp and a required minimum quality score of 20 [10]. Host reads were removed prior to assembly by mapping against the Aedes aegypti reference genome (NCBI assembly ID: GCF_002204515.2). rRNA was not specifically depleted during library preparation or bioinformatically removed, as the study aimed to capture total viral diversity. High-quality reads were assembled de novo into contigs using SPAdes (v3.15.5) in ‘careful’ mode [11]. For viral identification, the assembled contigs were compared against the RefSeq viral database (NCBI) using BLASTn (v2.14.0) with an e-value cutoff of 1 × 10^−5^, retaining the top five hits per query with a minimum alignment length of 100 nucleotides.

### 2.5. Phylogenetic Analysis

To determine the phylogenetic placement of the identified viruses, reference sequences for each viral species were retrieved from GenBank. Multiple sequence alignment was performed using the ClustalW algorithm in MEGA 12 software. Phylogenetic trees were reconstructed using the maximum likelihood method in MEGA 12 with the best-fit nucleotide substitution model [12]. Tree robustness was assessed with 1000 bootstrap replicates. In all phylogenetic figures, tip labels include the GenBank accession number, country of isolation, and year of isolation (where available; “n/a” indicates metadata not available in the original accession).

### 2.6. Data Visualization

All data processing and visualization were performed using Python (version 3.8+) [13], including handling and transformation using the Pandas library [14]. Static statistical plots, and the presence-absence heatmap, were generated using the matplotlib (version 3.5+) and seaborn (version 0.11+) libraries [15,16]. For the creation of interactive and hierarchical visualizations, the Plotly library (v6.4.0) for Python was used [17].

## 3. Results

**Figure 2:**
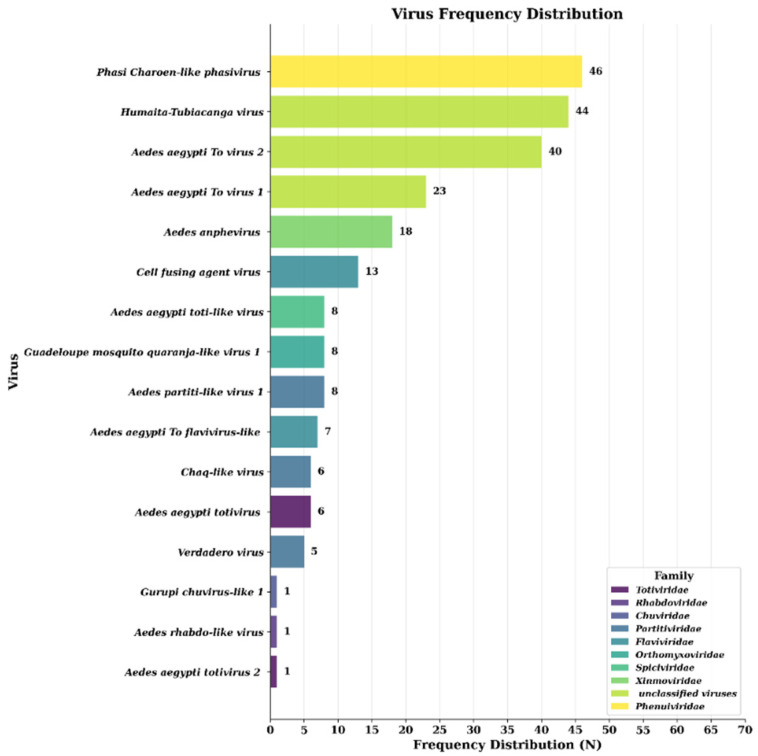
Distribution of mosquito-associated virus detections across Vietnamese provinces. Viruses are stratified in descending order of detection frequency, with color coding representing different viral families. Numerical labels adjacent to each bar indicate absolute detections (N) for each virus. The *x*-axis scale extends to 69, representing cumulative detection events across all viral species.

**Figure 3:**
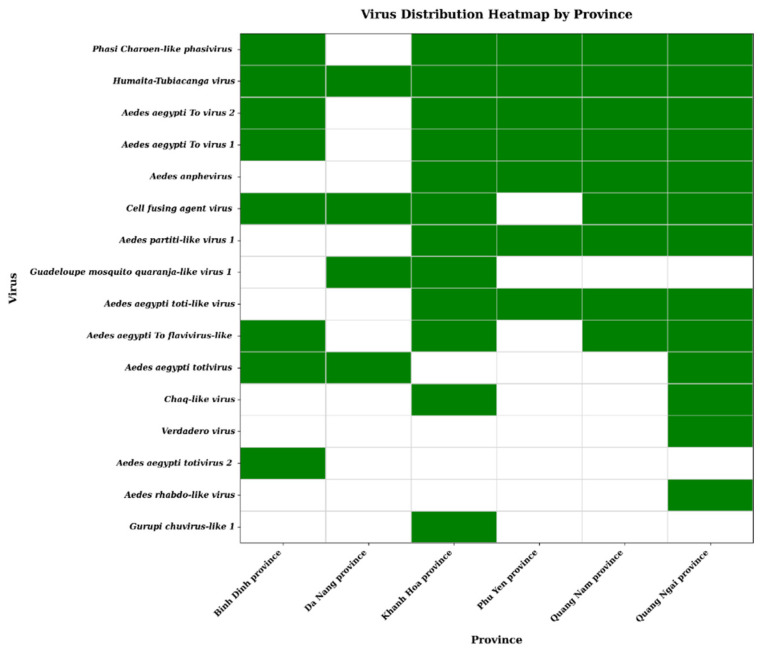
Geographic distribution of mosquito-associated viruses across Vietnamese provinces. Green cells indicate viral presence in the corresponding province; white cells denote absence. Viruses are stratified in descending order of detection frequency for comparative analysis.

A total of 69 NGS libraries were constructed and sequenced, resulting in 10.35 Gb of raw data, totaling 50.5 million reads, with an average of 732,000 raw reads per sample. After quality control, 6.89 Gb of clean data and 47.7 million high-quality reads remained, averaging 691,000 reads per sample. The negative control (no-template control) sequenced in parallel yielded no detectable viral sequences after bioinformatic analysis, confirming the absence of significant cross-contamination or reagent-derived viral signals. The percentage of PCR duplicates in clean data varied across samples, reaching up to 61.9%. From these, 940 viral contigs were obtained through de novo assembly from the 69 samples. The number of contigs associated with an individual virus within a sample was variable, reaching a maximum of 19. Following BLAST (v2.14.0) analysis against the viral database, the contigs were classified into nine viral families: *Totiviridae*, *Flaviviridae*, *Partitiviridae*, *Phenuiviridae*, *Orthomyxoviridae*, *Xinmoviridae*, *Spiciviridae*, *Rhabdoviridae*, and *Chuviridae*. The most prevalent viruses across samples were *Phasi Charoen-like phasivirus* (PCLV, *Phenuiviridae*), *cell fusing agent virus* (CFAV, *Flaviviridae*), and *Humaita-Tubiacanga virus* (HTV, unclassified), detected in 46, 38, and 30 pools, respectively (Figure 2). In total, 16 distinct insect-specific viruses (ISVs) were identified, including three with undefined taxonomic positions in the current ICTV taxonomy: *Aedes aegypti To virus 1*, *Aedes aegypti To virus 2*, and HTV (Figure 2 and Figure 3; Table S2). Classification as ISV was based on the absence of vertebrate host associations in BLAST searches, phylogenetic clustering with known insect-specific viruses, and their taxonomic affiliation with families predominantly associated with insect hosts [3,4,5].

Comparative analysis of metaviromic composition in dengue virus-infected (n = 69) and uninfected (n = 7) mosquito pools revealed both common features and substantial differences. As shown in Table S2, the core metavirome consisted of 11 viruses that were detected in both sample groups. This stable “core metavirome” includes widely distributed insect-specific viruses, such as CFAV (*Flaviviridae*) and PCLV (*Phenuiviridae*), along with several *Totiviridae* and *Partitiviridae* family representatives.

**Figure 4:**
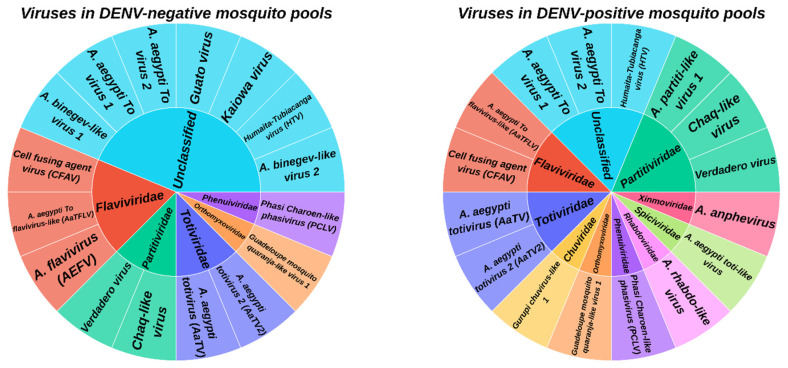
Distribution of mosquito viruses by family and DENV detection group. The diagram shows the hierarchical structure of viruses, grouped by families (inner circle) and individual viruses (outer circle).

However, a group of five viruses was detected only in DENV-negative pools (absent in DENV-infected mosquitoes): *Aedes flavivirus* (AEFV, *Flaviviridae*); *Guato virus*; *Kaiowa virus*; *Aedes binegev-like virus 1*; and *Aedes binegev-like* virus 2 (unclassified). Conversely, a unique group of five viruses was detected solely in DENV-positive pools. This group included representatives from families such as *Partitiviridae* (AePLV1), *Xinmoviridae* (*Aedes anphevirus*), *Spiciviridae* (*Aedes aegypti toti-like virus*), *Rhabdoviridae* (*Aedes rhabdo-like virus*), and *Chuviridae* (*Gurupi chuvirus-like 1*) (Figure 4, Table S2).

**Figure 5:**
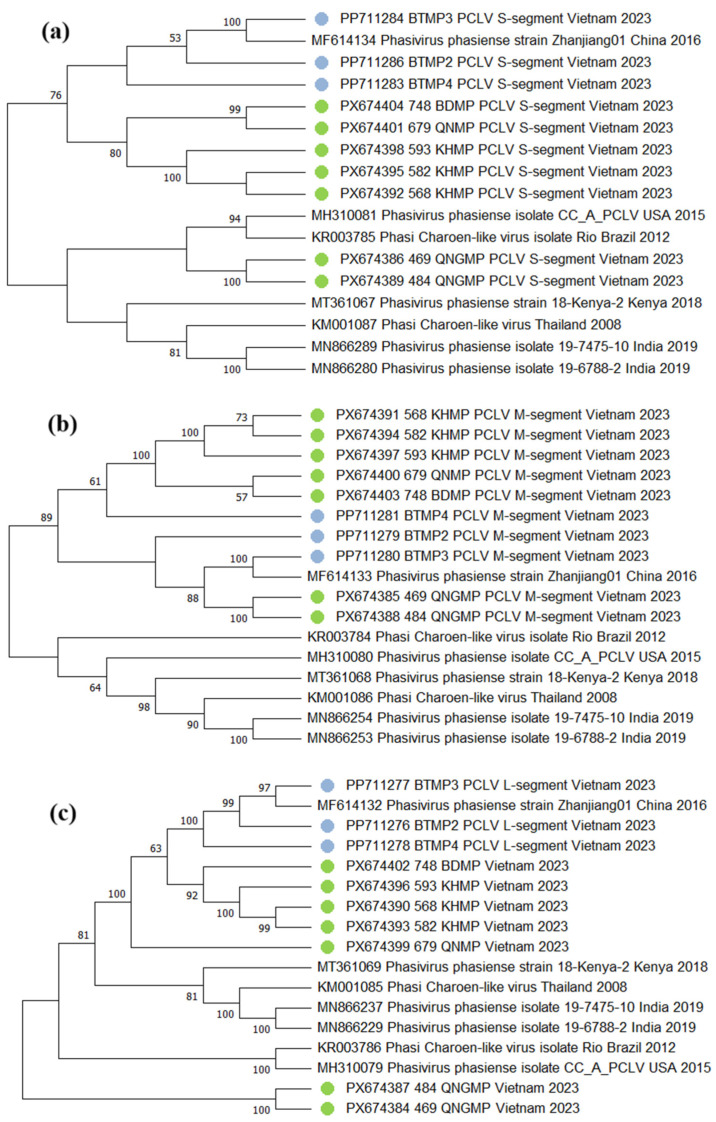
Phylogenetic tree of *Phasi Charoen-like phasivirus* (PCLV) genomic segments. Sequences obtained during the study are marked with green dots. Sequences from the previous study are marked with blue dots. Branches corresponding to partitions reproduced in less than 50% bootstrap replicates are collapsed. Where metadata (collection date or location) were not available in the GenBank record, "N/A" is indicated. (**a**) S segment. Evolutionary history inferred using the maximum likelihood method and Tamura-Nei model [18]. The analysis involved 17 nucleotide sequences. The final dataset contained 1335 bp. (**b**) M segment. Evolutionary history inferred using the maximum likelihood method and Tamura-Nei model [18]. The analysis involved 10 nucleotide sequences. The final dataset contained 3770 bp. (**c**) L segment. Evolutionary history inferred using the maximum likelihood method and general time-reversible model [19]. The analysis involved 10 nucleotide sequences. The final dataset contained 6682 bp.

Given its high prevalence (46/69 pools), prior evidence of reassortment in the region, and the documented capacity of insect-specific viruses to modulate arbovirus replication, we performed a detailed phylogenetic analysis of PCLV [5,6]. For PCLV (*Phenuiviridae*), seven near-complete sequences each were assembled for the L segment (RdRp), M segment (glycoprotein), and S segment (nucleocapsid). Sequences from these seven isolates were used to construct the phylogenetic trees for each segment (Figure 5). Isolates 469_QNGMP and 484_QNGMP form a stable cluster across all three segments with maximum support (100%). The KHMP isolates (568, 582, 593) form a stable cluster in all segments. By L segment, 748_BDMP clusters with KHMP. By M and S segments, it clusters with 679_QNMP. The phylogenetic position of 679_QNMP varies across segments. It should be noted that while the L and S segment trees are generally well supported, the M segment tree contains several nodes with bootstrap values below 70%. This may reflect higher genetic variability of the glycoprotein gene, limited phylogenetic signal, or possible recombination events.

**Figure 6:**
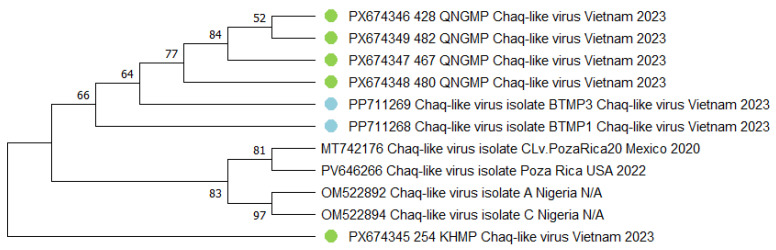
Phylogenetic tree of the *Chaq-like virus* fragment. The evolutionary history was inferred using the maximum likelihood method and Tamura-Nei model [18]. Branches corresponding to partitions reproduced in less than 50% bootstrap replicates are collapsed. This analysis involved eleven nucleotide sequences. There were a total of 730 bp in the final dataset. The sequences obtained in the current study are marked with green dots. Sequences from the previous study are marked with blue dots. Where metadata (collection date or location) were not available in the GenBank record, "N/A" is indicated.

Phylogenetic analysis revealed two contrasting genetic patterns among the *Chaq-like virus* isolates (Figure 6). Firstly, a group of four identical isolates (428, 467, 480, 482) was identified in the QNGMP pools (100% nucleotide sequence identity). This is an epidemiologically significant finding that most likely indicates their recent common origin and the active circulation of a single genetic variant of the virus within this specific mosquito population. The second key discovery involved the characterization of the 254_KHMP isolate, which stands in sharp contrast to the group described above. This isolate is distinguished by a significant genetic distance from all other studied sequences. Its basal position on the phylogenetic tree may reflect either a more ancient evolutionary lineage or, alternatively, greater genetic diversity within the *Chaq-like virus* family than is currently represented in public databases. This finding highlights the need for broader sampling to fully capture the diversity of these viruses in mosquito populations.

**Figure 7:**
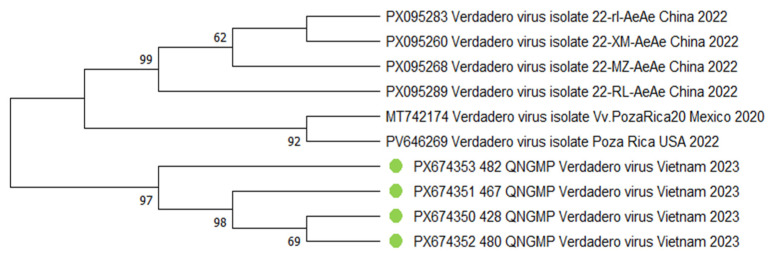
Phylogenetic tree of the *Varadero virus* fragment. The evolutionary history was inferred using the maximum likelihood method and Tamura-Nei model [18]. Branches corresponding to partitions reproduced in less than 50% bootstrap replicates are collapsed. This analysis involved eleven nucleotide sequences. There were a total of 1594 bp in the final dataset. The sequences obtained in the current study are marked with green dots. Where metadata (collection date or location) were not available in the GenBank record, "N/A" is indicated.

For the *Verdadero virus*, phylogenetic analysis revealed a structured organization within the cluster formed by isolates from the QNGMP mosquito population. All studied isolates form a single compact group, within which a certain hierarchy of genetic relationships is observed. Specifically, isolates 428_QNGMP and 480_QNGMP feature the highest degree of genetic similarity, forming a conserved pair. A sequential branching pattern is observed from this pair to other isolates, which exhibit a somewhat greater genetic distance (Figure 7).

**Figure 8:**
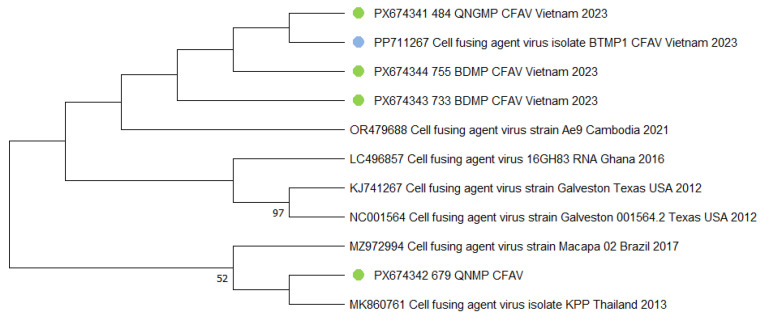
Phylogenetic tree of the *cell fusing agent virus* (CFAV) fragment. The evolutionary history was inferred using the maximum likelihood method and Kimura 2-parameter model [20]. Branches corresponding to partitions reproduced in less than 50% bootstrap replicates are collapsed. This analysis involved ten nucleotide sequences. There were a total of 370 bp in the final dataset. The sequences obtained in the current study are marked with green dots. Sequences from the previous study are marked with blue dots. Where metadata (collection date or location) were not available in the GenBank record, "N/A" is indicated.

For the CFAV, phylogenetic analysis revealed distinct clustering patterns. Isolate 484_QNGMP forms a direct pairing with PP711267, and it groups into a single cluster with samples 755_BDMP and 733_BDMP. This suggests a potential shared transmission chain within the local region. In contrast, isolate 679_QNMP features a different evolutionary pathway. It first clusters with an isolate from Thailand (MK860761), and this pair subsequently forms a larger cluster with a Brazilian isolate (MZ972994) (Figure 8).

**Figure 9:**
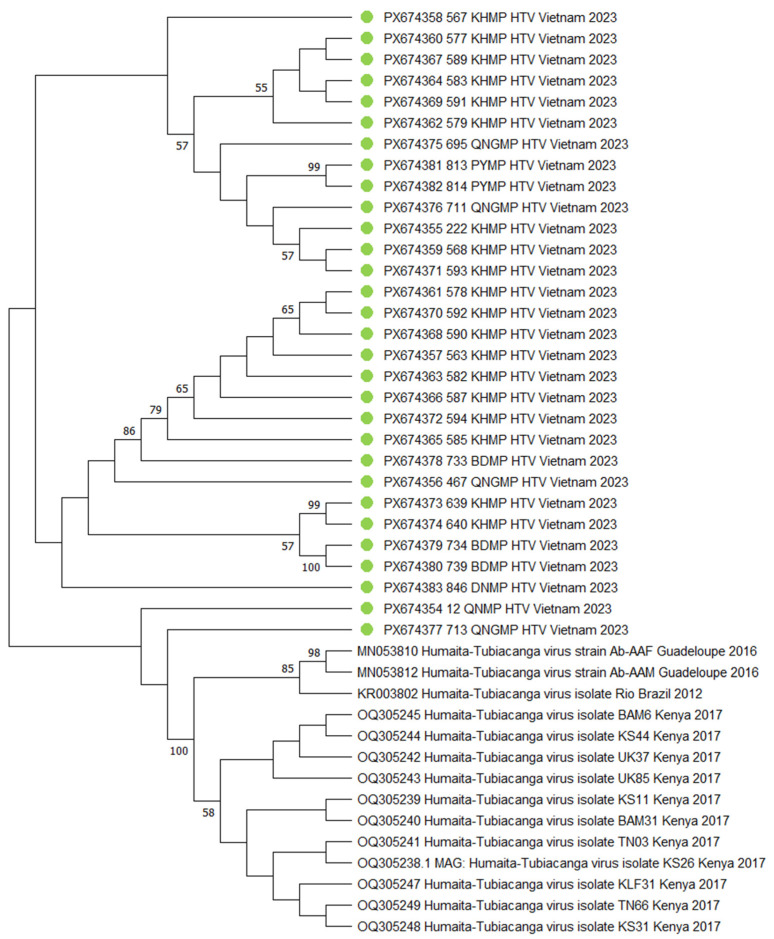
Phylogenetic tree of the *Humaita-Tubiacanga virus* (HTV) fragment. The evolutionary history was inferred using the maximum likelihood method and Tamura-Nei model [18]. Branches corresponding to partitions reproduced in less than 50% bootstrap replicates are collapsed. This analysis involved 14 nucleotide sequences. There were a total of 1395 bp in the final dataset. The sequences obtained during the study are marked green dots. Where metadata (collection date or location) were not available in the GenBank record, "N/A" is indicated.

The phylogenetic tree reveals a complex structure with two main evolutionary lineages containing field samples of HTV. The distribution of the isolates reflects an intertwining of geographical and genetic patterns. The first main branch predominantly comprises samples from KHMP locations, forming several subclusters: a tight group with isolates 583_KHMP_HTV and 591_KHMP_HTV; a cluster involving 577_KHMP_HTV and 589_KHMP_HTV; and a subgroup including 695_QNGMP_HTV and PYMP samples. Additionally, sample 567_KHMP_HTV occupies a basal position relative to the other KHMP isolates within this branch. The second main branch is characterized by greater diversity and includes samples from various geographical locations. Reference sequences form distinct clusters within the second main branch. Samples 12_QNMP_HTV and 713_QNGMP_HTV show closeness to these reference groups, while the majority of the other studied isolates form separate clusters (Figure 9).

**Figure 10:**
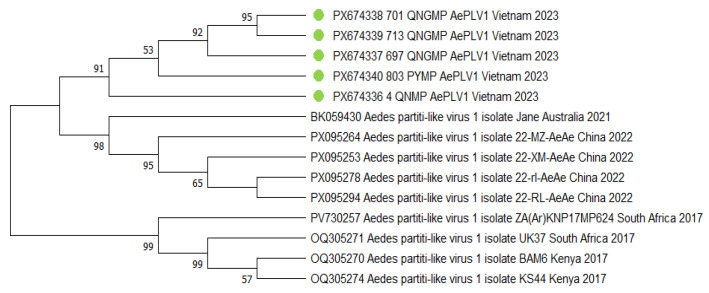
Phylogenetic tree of the *Aedes partiti-like virus 1* (AePLV1) fragment. The evolutionary history was inferred using the maximum likelihood method and Kimura 2-parameter model [20]. Branches corresponding to partitions reproduced in less than 50% bootstrap replicates are collapsed. This analysis involved ten nucleotide sequences. There were a total of 1382 bp in the final dataset. The sequences obtained in the current study are marked with green dots. Where metadata (collection date or location) were not available in the GenBank record, "N/A" is indicated.

Phylogenetic analysis of AePLV1 reveals a clearly defined divergence into two principal evolutionary lineages. The tree features a consistent separation: one clade is represented exclusively by the study samples from various geographical collection sites, while the other consists solely of reference sequences obtained from GenBank. This topological structure provides compelling evidence for significant genetic isolation of the isolates circulating in the study region. The studied samples form a compact monophyletic cluster, displaying a sequential hierarchical structure. The basal position within the cluster is occupied by samples 701_QNGMP_AePLV1 and 713_QNGMP_AePLV1, which form a stable group. Isolate 697_QNGMP_AePLV1 sequentially joins this pair, potentially indicating its derived origin. Subsequently, isolate 803_PYMP_AePLV1 joins the formed group, featuring a more divergent position. The branch is terminated by sample 4_QNMP_AePLV1, occupying the most derived position. The observed sequential branching suggests a directional pattern of viral evolution within the study region. The stepwise addition of isolates to the cluster may reflect the sequential spread of the virus between geographical locations. The stability of the clustering, and the monophyletic nature of the study isolate group, support their shared evolutionary origin and potential epidemiological linkage (Figure 10).

## 4. Discussion

This study represents the first comparative analysis of the metavirome of *Ae. aegypti* mosquitoes from Vietnam in the context of their dengue virus (DENV) infection status. Our analysis revealed a complex metaviromic structure and allowed us to hypothesize about potential interactions between resident insect-specific viruses (ISVs) and DENV. Our comparative analysis reveals distinct patterns of ISV association with DENV infection status in wild *Ae. aegypti* populations. A stable ‘core metavirome’ was observed across both groups, suggesting that certain ISVs (e.g., CFAV, PCLV) maintain persistent infections regardless of DENV presence. More strikingly, several ISVs were detected exclusively in either DENV-positive or DENV-negative mosquitoes, pointing to potential ecological and functional relationships that warrant experimental investigation. Their constant presence, irrespective of DENV infection status, suggests that these viruses form the foundation of the species-specific *A. aegypti* metavirome in the region, and they are likely in a state of stable equilibrium with the host. In contrast, the most intriguing aspect of our study was the identification of two ISV groups exclusively associated with one of the DENV statuses.

### 4.1. Viruses Associated with DENV Negativity

The detection of five viruses that were obtained only in DENV-negative pools allows us to hypothesize their potential antagonistic effect on DENV replication. This pattern is consistent with experimental studies demonstrating that some insect-specific flaviviruses can suppress arbovirus replication in mosquito cells [4,21,22]. For example, an isolate of Aedes flavivirus from Brazil was shown to inhibit replication of DENV and yellow fever virus in Aedes albopictus C6/36 cells [22]. It is known that viruses within the same family can compete for cellular resources or induce cross-reactive immunity [4,21,23,24,25]. Thus, it could be assumed that the presence of these ISVs creates an environment within the mosquito that is unfavorable for the establishment of DENV infection. However, such associations are not universal, and the mechanisms remain poorly understood.

### 4.2. Viruses Associated with DENV Coinfection

The identification of five viruses found exclusively in DENV-positive pools raises the possibility of synergistic interactions that warrant experimental validation. These were: *Aedes partiti-like virus 1* (AePLV1), *Aedes anphevirus*, *Aedes aegypti toti-like virus*, *Aedes rhabdo-like virus*, and *Gurupi chuvirus-like 1*. *Aedes anphevirus* has been shown in cell culture to modulate the antiviral response and enhance DENV replication under certain conditions, while AePLV1 belongs to the *Partitiviridae* family, members of which are known to persistently infect insects with limited pathogenicity [21]. Their presence may indirectly facilitate DENV replication, for instance, by suppressing insect antiviral pathways, thereby creating more favorable conditions for the pathogen [26,27]. These findings require further investigation, but provide a basis for targeted in vitro experimental studies to establish causal relationships in these interactions.

Phylogenetic analysis revealed distinct distribution patterns for the studied viral isolates. When interpreting the results, it is important to consider that reference nucleotide sequences available in open databases (NCBI) are still limited for some of these viruses. Geographic and temporal coverage data may also be insufficient. This uneven data coverage may have influenced the observed phylogenetic structure. As such, the following conclusions should be considered preliminary.

In this context, it can be hypothesized that processes of both local circulation and spatial spread of viruses may occur simultaneously in mosquito populations. A summary of geographic distribution and phylogenetic patterns for each virus is provided in Table S3. The most pronounced signs of possible local circulation are observed for PCLV and *Verdadero viruses*. For PCLV, the formation of clusters generally corresponding to the geographic origin of the isolates was noted. The small genetic distances within some of these clusters may indicate a close phylogenetic relationship of their isolates. Similar features were observed for the *Verdadero virus*, whose isolates form a compact group with minimal genetic distances, apparently suggesting stable circulation.

The data for *Chaq-like virus* may reflect a more complex dynamic. The presence of identical sequences among a number of isolates may indirectly indicate their active transmission, while the divergence of other isolates suggests their prolonged circulation under conditions of relative isolation.

More complex and ambiguous distribution patterns were identified for CFAV and HTV viruses. The observed intermingling of CFAV isolates from different collection sites, including the close relationship between some of them, could potentially result from a shared transmission chain. Of particular interest is the placement of isolate 679_QNMP, which clustered with strains from Thailand and Brazil. However, given the fragmentary nature of the available genetic data, this observation could be explained either by the global distribution of similar variants or by insufficient sample representativeness. The phylogenetic pattern for HTV also shows no strict geographical clustering. The co-clustering of isolates from different regions may be associated with vector migration processes, but also requires further verification with more extensive data.

The pattern observed for the AePLV1 virus deserves special attention. All its isolates form a single cluster, which is consistent with the hypothesis of active exchange within the region. The evolution and circulation of viruses in the mosquito populations of the studied region are characterized by multidirectional processes. The key pattern we observed was an association of certain ISVs with DENV infection status, alongside phylogenetic diversity. This may be partly a consequence of existing gaps in the genetic surveillance of these viruses. For more confident conclusions, and the construction of complete evolutionary scenarios in the future, an expansion of databases with new sequences covering wider geographic and temporal ranges will be required.

### 4.3. Comparison with Other Geographical Regions

Comparisons with metavirome studies from other geographical regions reveal both conserved and variable features. The core viruses identified here (e.g., PCLV, CFAV,) are frequently reported in *Ae. aegypti* worldwide, suggesting ancient associations and global dispersal [28,29,30,31]. For instance, studies from Thailand and Australia have demonstrated remarkable similarity in virome composition across geographically distant populations, with up to 16 insect-specific viruses shared between Bangkok and Cairns [28]. In contrast, the exclusive associations with DENV status observed in our study have not been consistently documented elsewhere. For instance, *Aedes anphevirus* is widespread globally, with distinct African, American, and Asia-Pacific lineages reflecting the historical expansion of its mosquito host, but its link to DENV infection has not been reported in field studies from the Americas or Africa [21]. This discrepancy may reflect regional differences in vector population genetics, DENV serotypes, or environmental factors. Alternatively, it could be due to the limited sample sizes of previous studies or the fact that most metavirome surveys have focused on virus discovery rather than status-based comparisons. Our findings underscore the need for multi-country standardized surveys to disentangle the ecological drivers of ISV-arbovirus interactions.

### 4.4. Limitations of the Study

We acknowledge several limitations. First, the use of mosquito pools rather than individual insects precludes an unambiguous determination of whether detected ISVs and DENV originated from a single mosquito or resulted from viral mixing from different individuals within the same pool. Second, the DENV-negative control group originated from a single province (Binh Thuan) and, despite being collected in the same year (2023), may not fully represent the metavirome background across all six provinces where DENV-positive mosquitoes were collected. Moreover, its small size (n = 7 pools) limits statistical power; thus, observed differences in viral presence/absence should be interpreted as descriptive associations rather than definitive conclusions. Third, while our conservative bioinformatics pipeline (stringent BLAST criteria, host read removal) ensured high-confidence viral detection, it may have underestimated highly divergent or very low-abundance viruses. The average sequencing depth (~0.73 million reads per pool) may also have precluded detection of the rarest viruses and complete genome assembly. Fourth, incomplete metadata (collection date/location) for some GenBank reference sequences and low bootstrap support at certain nodes (e.g., PCLV M segment) constrain phylogeographic interpretations and evolutionary inference; full-genome sequencing would help resolve these uncertainties. Therefore, this study is exploratory and aims to generate hypotheses for future controlled experimental studies on ISV-DENV interactions.

## Data Availability

The nucleotide sequences presented in this article have been deposited in GenBank under accession numbers PX674336–PX674404. The data will be made publicly available after the manuscript is accepted for publication.

## Supplementary Materials

The following supporting information can be downloaded at: https://www.mdpi.com/article/10.3390/v18040422/s1, Table S1: Samples collected by region; Table S2: Prevalence of insect-specific viruses in DENV-positive and DENV-negative *Aedes aegypti* mosquito pools; Table S3: Geographic distribution.
