# Supplementary material for: Aedes aegypti Mosquitoes from Central Vietnam Feature Specific Viromic Profiles Linked to Dengue Virus Coinfection
Source: Viruses. 2026 Mar 31;18(4):422. doi: 10.3390/v18040422 (PMC13119931; doi:10.3390/v18040422)
Supplement: Supplementary file 1 [file viruses-18-00422-s001.zip › Table S1.pdf]

Table S1. Samples collected by region.

Province	Sample IDs	Collection period (2023)	Total
**Khanh Hoa**	222_KHMP, 254_KHMP (Jan); 563_KHMP–594_KHMP (Jun); 639_KHMP, 640_KHMP (Nov)	Jan, Jun, Nov	26
**Phu Yen**	803_PYMP–832_PYMP (Nov)	Nov	17
**Quang Ngai**	428_QNGMP–484_QNGMP (Jun); 695_QNGMP–713_QNGMP (Nov)	Jun, Nov	14
**Quang Nam**	4_QNMP–14_QNMP (Jan); 679_QNMP (Nov)	Jan, Nov	5
**Binh Dinh**	733_BDMP–755_BDMP (Nov)	Nov	5
**Da Nang**	215_DNMP (Jan); 846_DNMP (Nov)	Jan, Nov	2
Total			**69**
