# Supplementary material for: Aedes aegypti Mosquitoes from Central Vietnam Feature Specific Viromic Profiles Linked to Dengue Virus Coinfection
Source: Viruses. 2026 Mar 31;18(4):422. doi: 10.3390/v18040422 (PMC13119931; doi:10.3390/v18040422)
Supplement: Supplementary file 1 [file viruses-18-00422-s001.zip › Table S2.pdf]

Table S2. Prevalence of insect-specific viruses in DENV-positive and DENV-negative *Aedes aegypti* mosquito pools.

Virus	Viral Family	DENV-negative Mosquito Pools	DENV-positive Mosquito Pools
**Viruses detected in both pool types**			
Aedes aegypti totivirus (AaTV)	*Totiviridae*	detected	detected
Aedes aegypti totivirus 2 (AaTV2)	*Totiviridae*	detected	detected
Cell fusing agent virus (CFAV)	*Flaviviridae*	detected	detected
Aedes aegypti To flavivirus-like (AaTFLV)	*Flaviviridae*	detected	detected
Verdadero virus	*Partitiviridae*	detected	detected
Chaq-like virus	*Partitiviridae*	detected	detected
Phasi Charoen-like phasivirus (PCLV)	*Phenuiviridae*	detected	detected
Guadeloupe mosquito quaranja-like virus 1	*Orthomyxoviridae*	detected	detected
Aedes aegypti To virus 1	Unclassified	detected	detected
Aedes aegypti To virus 2	Unclassified	detected	detected
Humaita-Tubiacanga virus (HTV)	Unclassified	detected	detected
**Viruses detected only in DENV-negative pools**			
Guato virus	Unclassified	detected	not detected
Aedes flavivirus (AEFV)	*Flaviviridae*	detected	not detected
Kaiowa virus	Unclassified	detected	not detected
Aedes binegev-like virus 1	Unclassified	detected	not detected
Aedes binegev-like virus 2	Unclassified	detected	not detected
**Viruses detected only in DENV-positive pools**			
Aedes partiti-like virus 1 (AePLV1)	*Partitiviridae*	not detected	detected
Aedes anphevirus	*Xinmoviridae*	not detected	detected
Aedes aegypti toti-like virus	*Spiciviridae*	not detected	detected
Aedes rhabdo-like virus	*Rhabdoviridae*	not detected	detected
Gurupi chuvirus-like 1	*Chuviridae*	not detected	detected
