# Supplementary material for: Aedes aegypti Mosquitoes from Central Vietnam Feature Specific Viromic Profiles Linked to Dengue Virus Coinfection
Source: Viruses. 2026 Mar 31;18(4):422. doi: 10.3390/v18040422 (PMC13119931; doi:10.3390/v18040422)
Supplement: Supplementary file 1 [file viruses-18-00422-s001.zip › Table S3.pdf]

Table S3. Geographic distribution

Virus	Provinces detected (n=number of positive pools)
Phasi Charoen-like phasivirus (PCLV)	Da Nang (1), Quang Nam (5), Quang Ngai (9), Binh Dinh (3), Phu Yen (3), Khanh Hoa (24)
Cell fusing agent virus (CFAV)	Da Nang (1), Quang Nam (3), Quang Ngai (3), Binh Dinh (4), Khanh Hoa (1)
Humaita-Tubiacanga virus (HTV)	Da Nang (2), Quang Nam (3), Quang Ngai (7), Binh Dinh (4), Phu Yen (2), Khanh Hoa (24)
Aedes aegypti To virus 1	Quang Nam (2), Quang Ngai (7), Binh Dinh (2), Phu Yen (4), Khanh Hoa (8)
Aedes aegypti To virus 2	Quang Nam (2), Quang Ngai (7), Binh Dinh (2), Phu Yen (12), Khanh Hoa (17)
Aedes anphevirus	Quang Nam (1), Quang Ngai (6), Phu Yen (6), Khanh Hoa (5)
Aedes partiti-like virus 1 (AePLV1)	Quang Nam (1), Quang Ngai (4), Phu Yen (1), Khanh Hoa (2)
Aedes aegypti To flavivirus-like (AaTFLV)	Quang Nam (1), Quang Ngai (1), Binh Dinh (1), Khanh Hoa (4)
Aedes aegypti toti-like virus (AaTLV)	Quang Nam (1), Quang Ngai (1), Phu Yen (3), Khanh Hoa (3)
Guadeloupe mosquito quaranja-like virus 1 (GMQLV1)	Da Nang (1), Khanh Hoa (7)
Aedes aegypti totivirus (AaTV)	Da Nang (1), Quang Ngai (2), Binh Dinh (2)
Chaq-like virus	Quang Ngai (5), Khanh Hoa (1)
Verdadero virus	Quang Ngai (5)
Aedes rhabdo-like virus (ARLV)	Quang Ngai (1)
Aedes aegypti totivirus 2 (AaTV2)	Binh Dinh (1)
Gurupi chuvirus-like 1	Khanh Hoa (1)
Aedes aegypti To virus 1	Quang Nam (2), Quang Ngai (7), Binh Dinh (2), Phu Yen (4), Khanh Hoa (8)
Aedes aegypti To virus 2	Quang Nam (2), Quang Ngai (7), Binh Dinh (2), Phu Yen (12), Khanh Hoa (17)
Aedes anphevirus	Quang Nam (1), Quang Ngai (6), Phu Yen (6), Khanh Hoa (5)
Aedes partiti-like virus 1 (AePLV1)	Quang Nam (1), Quang Ngai (4), Phu Yen (1), Khanh Hoa (2)
Aedes aegypti To flavivirus-like (AaTFLV)	Quang Nam (1), Quang Ngai (1), Binh Dinh (1), Khanh Hoa (4)
Aedes aegypti toti-like virus (AaTLV)	Quang Nam (1), Quang Ngai (1), Phu Yen (3), Khanh Hoa (3)
